# The impact of training on farmers’ human capital accumulation in rural China

**DOI:** 10.1371/journal.pone.0340885

**Published:** 2026-01-27

**Authors:** Junxia Zeng, Lei Wan, Wenjin Long

**Affiliations:** 1 Rural Development Institute, Chinese Academy of Social Sciences, Beijing, China; 2 Central Agricultural Broadcasting and Television School, Beijing, China; 3 College of Economics and Management, China Agricultural University, Beijing, China; Yunnan University, CHINA

## Abstract

Training represents a crucial pathway for enhancing farmers’ human capital. China currently invests over 2 billion CNY (310 million USD) annually to train nearly a million professional farmers. While previous studies have primarily focused on the direct effects of training on production and income, there is no consensus on how training specifically contributes to farmers’ human capital accumulation. This study employs panel survey data from 753 farmers across four Chinese provinces collected in 2016 and 2019, utilizing Propensity Score Matching-Difference in Differences (PSM-DID) methodology to examine the impact of farmer training on participants’ human capital. The empirical analysis reveals that farmers who participated in professional training programs experienced significant improvements in their explicit human capital, demonstrated by increases of 6.76% and 13.30% in obtaining national vocational qualification certificates and farmer technical staff titles, respectively. Additionally, their internalized human capital showed marked enhancement, with increases of 21.03% and 11.29% in utilizing digital platforms such as WeChat groups and Douyin (or Kuaishou) for agricultural knowledge and skill acquisition. These findings suggest that China should continue prioritizing farmers’ human capital development, particularly through professional training programs, as a key strategy for promoting agricultural transformation and rural revitalization.

## Introduction

Since its introduction in the 1960s, human capital theory has become a crucial theoretical framework for explaining economic growth and income distribution. The theory emphasizes the vital role of education and training in enhancing individual productivity, asserting that investments in human capital can significantly improve workers’ skill levels and earning capacity, thereby increasing social welfare. In agriculture, human capital is particularly crucial as modern agricultural development increasingly depends on farmers’ ability to master new technologies, knowledge, and business concepts. The relatively low level of human capital among farmers is a global phenomenon existing not only in developing countries like China but also in developed countries where farmers typically have the lowest levels of education among all occupational groups [1]. This primarily stems from the general trend of highly skilled labor migrating to non-agricultural sectors and the relatively low efficiency of global agricultural production [2]. Therefore, improving farmers’ human capital through education and training to increase productivity and income is of great significance [3].

As a major agricultural producer in the world, China had 168.82 million agricultural workers in 2023, accounting for 22.8% of total employment. However, human capital among Chinese farmers remains relatively low compared to other sectors: only 6.5% of agricultural workers have high school education or above, 34.4 percentage points below the national average; 41.9% have middle school education, 3.5 percentage points above the national average; and 51.5% have primary school education or below, 30.8 percentage points higher than the national average [4]. Given the low educational attainment of agricultural workers and the difficulty in changing their formal education levels on a large scale, training has been viewed as the primary means of improving human capital among China’s existing agricultural workforce. However, Chinese farmers have historically had limited access to educational and skill training opportunities, which has constrained agricultural productivity growth and farmers’ income improvement. To address this issue, the Chinese government has intensified efforts to promote farmer education and training in recent years. In 2014, the Ministry of Agriculture and the Ministry of Finance jointly launched the New Professional Farmer Cultivation Project and invested 1.1 Billion CNY (177 million USD) in farmer training; by 2017, the professional farmer cultivation project had fully covered 2,027 agricultural counties (districts, farms) nationwide, training about 700 thousand people annually. By 2022, the total investments on farmer training exceeding 15.9 Billion CNY (278 million USD) [5]. The number of trained farmer reached more than 9 million during 2012–2024 [6]. As a strategic public policy intervention aimed at enhancing farmers’ human capital and subsequently improving agricultural productivity and rural household income levels, farmer training programs warrant systematic empirical investigation into their implementation effectiveness and socioeconomic impacts.

Despite the substantial government investment and large participation numbers in farmer training programs, empirical research on their effectiveness remains relatively limited. From international experience, while most studies demonstrate the positive effects of farmer training [7–9], some research has found that farmer training has no significant impact on trained farmers [10], or only partial significant effects [11], or no spillover effects on other farmers [12], which may be related to factors such as the degree of matching between farmer training and agricultural production skills, the quality of course content, and training duration [12]. Furthermore, many studies on farmer training effects focus on the changes in production, profit, and social domains brought about by training, concentrating primarily on short-term changes [9]. However, changes in human capital are the most direct and fundamental changes from farmer training, and it is precisely because of changes in human capital that other possible production, profit, and social changes are triggered [13]. The policy intention of farmer training is to improve farmers’ human capital levels, and only by relying on the improvement of farmers’ human capital levels can agricultural production efficiency and farmers’ agricultural income be fundamentally enhanced.

This study aims to systematically evaluate the impact of farmer training on human capital accumulation. Specifically, this research divides human capital into two dimensions: explicit human capital (including knowledge, skills, and certifications) and internalized human capital (including learning ability and information acquisition capacity). Using panel data from 753 farmers across four provinces collected 2019, we employ the Propensity Score Matching-Difference in Differences (PSM-DID) method to identify the causal effects of training. The empirical analysis reveals that farmer training significantly enhanced participants’ explicit human capital, as evidenced by increased rates of obtaining national vocational qualifications and agricultural technician titles. Simultaneously, internalized human capital showed marked improvement, with trained farmers demonstrating enhanced ability to utilize digital resources for agricultural knowledge and skill acquisition. These findings further validate the positive role of training programs in farmers’ human capital accumulation.

Compared to previous research, this study’s main contributions are threefold: First, while previous studies often assume that training can enhance farmers’ human capital and subsequently affect household production behavior, production efficiency and income, many farmer training programs actually suffer from poor targeting, inadequate instructors, and substandard training content, often resulting in superficial implementation with limited effectiveness. Therefore, it is necessary to investigate whether training can indeed improve farmers’ human capital as expected. This study focuses on the effects of farmer training on human capital, particularly the long-term effects of training, thereby better helping to understand the changes in production behavior and economic income found in previous research. Second, the measurement of post-training human capital requires further exploration, as most literature approaches this from a certification perspective, overlooking the long-term effects of training on improving farmers’ learning capabilities. This study adopts more objective indicators, simultaneously focusing on changes in farmers’ explicit and internalized human capital, enriching research on farmers’ human capital. Third, most previous studies use cross-sectional data, making it difficult to control for individual fixed effects and time trends, potentially leading to endogeneity issues that affect the credibility of results. The methods used in this study are more conducive to obtaining unbiased results of the impact of farmer training on human capital.

## Theoretical analysis and research hypotheses

### The multiple impact of farmer training programs on farmers

Farmer training programs have demonstrated significant positive effects on agricultural production and technology adoption. Studies have shown that farmers who participate in farmer-field-school (FFS) programs have significantly more knowledge about integrated pest management (IPM) practices than those in the nonparticipant comparison group [8,14]. These training initiatives help improve farmers’ mastery of complex knowledge [15], with farmers with higher knowledge levels demonstrating a tendency to reduce pesticide use [12]. Training programs are crucial for disseminating new agricultural technologies [16], significantly increasing farmers’ cognition and adoption of new agricultural technologies [17] and improved cultivation practices [18]. In Tanzania, training and demonstration programs increase the adoption rate of rice production technologies by up to 64.4% [19]. Farmers participating in training not only acquired production knowledge but also adopted many promoted new agricultural practices [20], with knowledge and skills gained from training being effectively applied to the cultivation of multiple crops [21]. Research has demonstrated that participation in potato technology training led to improvements in both farming techniques and potato yields among farmers in Ecuador [22], while training programs increased farmers’ adoption of constructing bunds and planting in rows, with significant positive impacts on rice yields [18].

The economic benefits of farmer training programs are well-documented, with substantial improvements in income, productivity, and welfare outcomes for participating farmers. Farmers who participated in rice technology training achieved significant improvements in both rice profits and yields [18], with trainees on plots where new technologies were adopted achieving rice yields and profits that were approximately 1.3-1.8 tons and 119–137 USD per hectare higher than on other plots, respectively [23]. Farmers participating in Farmer Field Schools in East Africa increased their agricultural income by 61% and improved overall crop productivity [24]. Through participation in Farmer Field Schools, agricultural households increased their real income per worker by approximately 60–160 USD over two years on average, which was equivalent to or even exceeded the average income per worker before the implementation of the training program [20]. Training received by farm families, their human capital, and their use of innovations improved household farm total factor productivity and farm equity [25]. Beyond income improvements, farmers’ participation in training and education has significantly reduced their poverty incidence rates [26] and relative economic poverty levels [27], and improved food security conditions among small-scale farmers [11,28].

Farmer training programs generate positive spillover effects that extend beyond direct participants to benefit broader rural communities and social networks. Case studies have confirmed that Farmer Field Schools strengthen social cohesion among farmers and enhance individual social skills through establishing new networks for knowledge exchange and support, promoting group formation, and improving farmers’ confidence and capacity to work more effectively in groups [29]. Furthermore, practical training programs have demonstrated improvements in organizational functioning through “enhanced inter-organizational relations” and “improved farm input services” [30]. Research indicates that farmer training impacts not only participants but also generates positive effects on non-participants, with training enhancing the adoption rates of improved technologies and productivity among non-participants through spillover effects [31], while household agricultural knowledge and food security are also improved [32]. Trained farmers are capable of enhancing neighboring farmers’ knowledge and practices regarding integrated pest management and pesticide handling [33], and educating and training other farmers to reduce the use of highly hazardous pesticides [34].

## Research hypotheses

In human capital theory, training participation represents a crucial pathway for human capital enhancement, with farmer training serving as a direct investment in agricultural human capital [35]. Through structured training programs, farmers acquire comprehensive knowledge spanning modern cultivation techniques, mechanization practices, sustainable production methods, digital agriculture, market management principles, and entrepreneurial competencies. For new agricultural entities, this training particularly enriches understanding of modern scale agricultural operations. Exposure to innovative technologies, varieties, equipment, and methodologies directly augments farmers’ human capital, enhancing their production capabilities and facilitating improved agricultural outcomes and income generation.

Training programs foster social capital formation, which subsequently reinforces human capital accumulation through information-sharing mechanisms. The enhanced social capital generates an important “information effect” that facilitates knowledge sharing, reduces information acquisition costs [36], and addresses information asymmetries. Regarding human capital development specifically, social capital promotes recognition of human capital’s value and encourages reinvestment behaviors through this “information effect” [37].

Existing research has primarily focused on specific agricultural knowledge or skill acquisition outcomes [14,16,38]. Studies examining broader human capital effects often rely on subjective self-reported measures, such as changes in “business management capabilities”, “information technology proficiency” and “innovation capacity” [15]. However, these abstract metrics present measurement challenges and potential perception biases.

Overall, the literature has generally emphasized specific agricultural competencies or operational capabilities rather than comprehensive vocational development. Modern agriculture demands increasingly sophisticated, specialized, and professional competencies, reflected in the evolution of training programs toward more systematic and comprehensive approaches [39]. Training impacts on professional knowledge and explicit human capital can be measured through objective indicators such as national vocational qualifications and technical titles, which reflect comprehensive occupational and industry-specific competencies.

Current research inadequately addresses training’s influence on internalized human capital – the learning capacity, motivation, and disposition that distinguish it from explicit knowledge and skills. This internalized component is particularly crucial in the Internet+ era, where agricultural knowledge rapidly evolves alongside digital integration [40]. Focusing solely on specific knowledge transfer in traditional settings may result in skill obsolescence. Effective training must cultivate broader learning capabilities (especially digital literacy) and sustainable learning motivation to ensure continuous human capital development. Modern training programs increasingly leverage digital platforms, enabling participants to access knowledge, market information, and professional networks through smartphones and social media platforms, fostering collaborative learning environments.

Based on the above analysis, farmer training demonstrates positive effects on participants’ human capital development, leading to the following research hypotheses:

H₁: Farmer training enhances participants’ explicit human capital (comprehensive knowledge and skills).

H₂: Farmer training improves participants’ internalized human capital (independent learning capacity and information acquisition capabilities).

## Materials and methods

### Data source

The data for this study is derived from a specialized survey evaluating professional farmer training effectiveness, conducted by the Central Agricultural Broadcasting and Television School (Farmer Science and Technology Education and Training Center of the Ministry of Agriculture and Rural Affairs) under the Department of Science, Technology, and Education of the Ministry of Agriculture and Rural Affairs in October 2020. The Central Agricultural Broadcasting and Television School is a specialized institution for farmer education and training in China. The school operates in virtually all provinces and undertakes more than half of the nationwide farmer training tasks. Since 2012, the professional farmer training program has primarily targeted new types of agricultural business entities, including large-scale farmers, family farm owners, key members of farmer cooperatives, and agricultural enterprise managers. Participants must demonstrate a certain level of production and leadership ability. The training curriculum includes modules on modern agricultural production techniques, agricultural business management, agricultural product marketing, and agricultural policies and regulations. The program typically lasts 15 days.

Following stratified random sampling principles, the survey selected one province from each of China’s four major regions: Jiangsu (East), Hubei (Central), Yunnan (West), and Heilongjiang (Northeast). Within each province, counties were ranked by GDP per capita and divided into high, medium, and low-level groups, with one county randomly selected from each group. This resulted in three sample counties per province (except Heilongjiang with two), totaling 11 counties across four provinces. In each county, 40 villages were randomly selected (or all villages if fewer than 40). From each village, 1–2 trained farmers were selected, with an equal number of untrained farmers matched from the same village, controlling for similarities in age, education, and production scale. Trained farmers were those who participated in the 2017 national new professional farmer training project, including Modern Young Farmer Development, New Agricultural Management Entity Leaders, Agricultural Manager Development, and Modern Entrepreneurship and Innovation Youth Development. Untrained farmers were those whose households had received no agricultural production and operation-related training.

The sampling scheme yielded 832 surveyed farmers, with 811 valid questionnaires (97.48% effective rate). Pure livestock farmers (58 individuals, 7.15%) were excluded to meet PSM method matching requirements. The final research sample comprised 753 farmers across 4 provinces, 11 counties, 76 townships, and 369 villages, including 381 trained and 372 untrained farmers.

The survey encompassed basic household information, training participation, pre- and post-training human capital, social capital, production and operation, family income, and other comparative characteristics. Given the extended cycle of agricultural production and the time required for training effects to manifest, the survey established three temporal data points: 2016 (pre-training), 2017 (training implementation), and 2019 (post-training), spanning a three-year interval.

### Variable selection

This study examines the impact of farmer training on human capital, specifically analyzing the differential effects between training participants (participation = 1) and non-participants (participation = 0) on farmers’ human capital development. Based on the classic human capital theories [35,41], as well as research on agricultural human capital and farmer learning behavior [41,42], we use two types of human capital in the analysis: Explicit human capital and internalized human capital.

Explicit human capital variables primarily measure farmers’ professional and comprehensive knowledge and skills, assessed through two indicators: attainment of national vocational qualification certificates (obtained = 1, not obtained = 0) and farmer technical titles (obtained = 1, not obtained = 0). The acquisition of national vocational qualifications or technical titles represents a significant milestone in professional maturity and serves as a key metric for stratified evaluation of practitioners’ human capital levels, enabling scientific and precise incentivization. Obtaining such certifications or titles indicates professional recognition of an individual’s comprehensive capabilities and human capital level within the industry [43]. The National Vocational Qualification Certificate system is a vocational skills evaluation and certification system that was established in China. It aims to standardize vocational skills, improve labor quality, and establish a unified evaluation mechanism for skilled personnel. The National Vocational Qualification Certificate standards specify the theoretical knowledge and operational skills required by practitioners, based on the content of their professional activities. Certificates are divided into five levels, ranging from entry-level worker to senior technician, with each level corresponding to different skill and knowledge requirements. Vocational qualifications for farmers mainly relate to agricultural technology occupations, such as crop protection technician, animal disease prevention and control technician, agricultural machinery repairer and agricultural manager. Some farmers who have switched to agriculture may have obtained other types of vocational qualification certificate prior to doing so. However, the proportion of farmers who obtain agricultural-related vocational qualification certificates is very low, and farmers rarely receive government incentives for doing so. Farmer Technical Titles provide a comprehensive evaluation of farmers’ technical and professional abilities, offering a more accurate reflection of their human capital. The criteria for evaluating these titles mainly include professional technical ability, production and management levels, and leadership and exemplary roles. The purpose is to encourage farmers to improve their technical skills and enhance their sense of professional honor. The evaluation covers multiple fields, including planting, breeding, processing agricultural products, and providing agricultural services. There are three levels: junior, intermediate, and senior. Farmers who obtain a title may receive cash rewards, project funding and honorary titles. Overall, National Vocational Qualification Certificates have a broader scope of application than Farmer Technical Titles, which are specifically for farmers. While vocational qualification certificates focus on the standardized evaluation of operational skills, technical titles emphasize a comprehensive technical level. Nevertheless, both are forms of human capital certification that contribute to improving farmers’ professional level.

Internalized human capital variables assess farmers’ learning ability and interest, particularly within the context of the Internet+ era. As network technology advances and internet economy increasingly integrates with agriculture, digital literacy has become a crucial component of farmers’ human capital affecting their production and lifestyle [40]. In the digital age, farmers need to be able to use smartphones and should continuously update their knowledge and skills using internet tools such as WeChat, Douyin and agricultural information apps. They must strengthen their self-learning awareness, cultivate digital thinking abilities, and promote the transformation and innovative application of knowledge. Therefore, “independent learning capacity” and “information acquisition capabilities” are important indicators of “internalized human capital”. Learning through digital platforms such as WeChat and Douyin has significantly improved farmers’ internalized human capital. Therefore, we measure the internalized human capital by the adoption of digital platforms for independent agricultural learning (yes = 1, no = 0), including WeChat groups and live streaming platforms like Douyin or Kuaishou. WeChat groups have established a continuous learning network based on training programs. Training organizers establish professional learning groups for each class and invite experts to join these groups in the long term. These experts regularly share cutting-edge technical information, innovative methods and industry trends. This “expert-farmer” dialogue model breaks the time and space constraints of traditional training, enabling real-time knowledge transfer. When farmers encounter specific production or management problems, they can seek help from experts and peers by providing text descriptions or images to obtain targeted solutions. Furthermore, a mutual learning network has formed among farmers within the group, who actively share information on superior varieties, expand sales channel resources and exchange project cooperation opportunities. This peer learning effect significantly enhances the speed at which knowledge is disseminated and applied. Douyin, a popular social platform for sharing short videos, is widely used by Chinese farmers and has become an important means of disseminating agricultural knowledge. The platform has attracted many professional agricultural technology bloggers who create videos based on seasonal agricultural production techniques, providing farmers with timely and practical technical guidance. This “fragmented, visualized and contextualized” learning method aligns more closely with farmers’ cognitive characteristics and learning habits. Douyin’s algorithmic recommendation mechanism can accurately suggest relevant agricultural content based on farmers’ interests and production needs, greatly expanding their learning resources. The platform overcomes the time and space limitations of traditional learning, enabling farmers to access the information they require at any time and in any location. This flexibility and convenience creates more opportunities for independent learning, effectively improving farmers’ ability to continuously learn and update their knowledge.

The study controls for various factors potentially influencing farmer training and human capital levels, including: (1) Individual characteristics: gender (female = 1, male = 0), age, education level (primary school and below = 1, middle school = 2, high school/vocational education = 3, junior college = 4, undergraduate and above = 5); (2) Family characteristics: internet access (yes = 1, no = 0), land operating area; (3) Village characteristics: distance to county town; (4) Provincial dummy variables. Table 1 presents the baseline data from 2016, before the implementation of new professional farmer training: 4.8% of respondents held national vocational qualification certificates, 7.2% had farmer technical titles, 25.8% used WeChat for agricultural learning, and 12.9% utilized platforms like Douyin or Kuaishou for knowledge acquisition. The sample comprised 74.2% male farmers, with an average age of 45.4 years. Educational attainment was predominantly middle school level, with 50.7% having middle school education or below, and 34.3% having high school education, indicating generally low educational levels. Internet access was present in 94.7% of households, suggesting widespread basic connectivity. The average family land operation area was 51.9 mu (3.46 hectares), with larger per-mu areas in Heilongjiang and Jiangsu provinces.

**Table 1:** Variable explanation and descriptive statistical analysis.

Category	Variables	Description	Mean	Standard deviation
Explicit human capital	Whether obtained national vocational qualification certificate	Yes = 1, No = 0	0.048	0.214
	Whether obtained farmer technical staff title	Yes = 1, No = 0	0.072	0.258
Internalized human capital	Whether used WeChat groups to learn agricultural knowledge and skills	Yes = 1, No = 0	0.258	0.438
	Whether used Douyin, Kuaishou, and other live streaming platforms to learn agricultural knowledge and skills	Yes = 1, No = 0	0.129	0.335
Individual characteristics	Gender	Male = 1, Female = 0	0.742	0.438
	Age	Unit: Years	45.422	9.05
	Education level	Primary school and below=1; Middle school = 2; High school = 3; Junior college = 4; Undergraduate and above=5	2.627	0.875
Family characteristics	Whether has internet	Yes = 1, No = 0	0.947	0.224
	Land operating area	mu*	51.95	103.935
Village characteristics	Distance from village to county town	Kilometers	33.179	34.633
Provincial dummy variables	Jiangsu	Yes = 1, No = 0	0.286	0.452
	Hubei	Yes = 1, No = 0	0.316	0.465
	Heilongjiang	Yes = 1, No = 0	0.248	0.432
	Yunnan	Yes = 1, No = 0	0.15	0.357

The observation value for each variable is uniformly 753 persons, with baseline data collection time in 2016.

* 1 mu is approximately equal to 0.0667 hectares, and 1 hectare equals 15 mu.

## Method

This study employs Propensity Score Matching combined with Difference-in-Differences (PSM-DID) to evaluate the human capital effects of farmer training, considering the characteristics of policy implementation, farmer participation, and human capital assessment requirements.

The Difference-in-Differences (DID) method, commonly used in policy evaluation, examines specific human capital variables, such as vocational qualification certificates, to assess the impact of professional farmer training. The Difference-in-Differences (DID) method, commonly used in policy evaluation, examines specific human capital variables, such as vocational qualification certificates, to assess the impact of professional farmer training. The method compares changes in outcomes between two time points (t = 0 for pre-training and t = 1 for post-training) for both trained (*D*_*i*_ = 1) and untrained (*D*_*i*_ = 0) groups. The difference between these two differences (Δ*Y*_*t*_ – Δ*Y*_*c*_) represents the policy intervention effect. The Average Treatment Effect on Treated (ATT) can be calculated through this double differentiation, expressed as:


$$\tau_{ATT\;}^{DID}(X_{it})=\;\text{E}[Y_{it}(\text{1})-Y_{it}(\text{0})|D_{i}=\text{1},\;\text{t}=\;\text{1}=\;\beta_{\text{3}}$$ (1)

The DID econometric model used to estimate ATT is:


$$Y_{it}\;=\;\beta_{\text{0}}\;+\;\beta_{i}D_{\text{1}}\;+\;\beta_{\text{2}}t\;+\;\beta_{\text{3}}(D_{i}\;\times \;t)\;+\;\beta_{\text{4}}X_{it}+\varepsilon_{it}$$ (2)

Where *D*_*i*_ indicates training group membership (1 = trained, 0 = untrained), t represents the policy implementation year dummy variable (1 represents 2019), β_*3*_ represents the training effect on human capital variables, and $X_{it}$ represents covariates including individual, family, village characteristics, and provincial dummy variables.

While DID doesn’t require homogeneity between groups before training, it assumes both groups follow the same change trend (“Parallel Trends” assumption). However, since training participation is self-selected rather than randomly assigned, systematic differences likely exist between groups, potentially violating this assumption and leading to biased results if using DID alone.

Therefore, this study implements PSM-DID to address potential self-selection bias. This combined approach leverages the strengths of both methods, first using PSM to construct more homogeneous control and treatment groups by controlling for observable variable differences, then applying DID to estimate the treatment effect ATT based on two-period data and the common trend assumption. The propensity score function P($X_{it}$) = Pr(*D*_*i*_ = 1|$X_{i}$) represents the probability of farmer observation in 2019 given observable characteristics X, replacing $X_{it}$ in equation (1). A probit model is used for propensity score calculation. The matching process selects samples within the common support region (S), matching each trained farmer with one or more similar untrained farmers using kernel matching with a 0.06 bandwidth. Balance tests are conducted to ensure result reliability and stability.

## Results

### Descriptive statistics

Table 2 illustrates the human capital variable values for both trained and untrained farmer groups before and after training. The data reveals that trained group farmers consistently maintained higher values across all human capital variables in both periods, with the disparity between groups notably expanding post-training.

**Table 2:** Changes in human capital before and after training.

Human capital	Untrained group		Trained group		Difference between before and after 3 years of training (2019−2016)		
	2016	2019	2016	2019	Untrained group	Trained group	Difference between groups
	Mean (SD)	Mean (SD)	Mean (SD)	Mean (SD)	Mean	Mean	Mean
Percentage of obtaining the following certificates or titles (%)							
National vocational qualification certificate	0.27 (5.18)	2.15 (14.53)	9.19 (28.92)	17.59 (38.12)	1.88***	8.4***	6.52***
Farmer technical staff title	2.15 (14.53)	3.76 (19.06)	12.07 (32.62)	26.77 (44.34)	1.61**	14.7***	13.09***
Percentage of using the following network means to learn agricultural knowledge and skills (%)							
WeChat groups	16.94 (37.56)	31.72 (46.6)	34.38 (47.56)	75.07 (43.32)	14.78***	40.69***	25.91***
Douyin, Kuaishou, and other live streaming platforms	9.14 (28.86)	37.1 (48.37)	16.54 (37.2)	62.2 (48.55)	27.96***	45.66***	17.7***
Sample size	372		381		372	381	753

T-statistical test significance level, ***, **, * represent 1%, 5%, 10% significance levels respectively.

Pre-training data shows that trained group farmers had higher rates of national vocational qualification certificates (9.19% vs. 0.27%) and farmer technical titles (12.07% vs. 2.15%) compared to the untrained group. Similarly, trained farmers demonstrated greater utilization of digital learning platforms, with 34.38% using WeChat groups (compared to 16.94% for untrained farmers) and 16.54% using Douyin, Kuaishou, and other streaming platforms (compared to 9.14%). Despite efforts to match trained and untrained farmers based on individual, family, and agricultural operation characteristics, systematic differences persisted, primarily due to trained farmers typically being professional operators with larger-scale production compared to general farmers in the untrained group. This underlying difference necessitates the application of rigorous research methods to isolate training-induced changes from systematic variations.

Post-training measurements revealed increased human capital variables across both groups, with trained farmers showing significantly higher growth rates. Their acquisition of national vocational qualification certificates and technical titles rose to 17.59% and 26.77% respectively (increases of 8.40% and 14.70%), while untrained farmers showed modest increases of 1.88% and 1.61%. Digital platform usage among trained farmers increased substantially: WeChat group utilization reached 75.07% (40.69% increase) and streaming platform usage rose to 62.20% (45.66% increase), compared to smaller increases among untrained farmers (14.78% and 27.96% respectively). These changes were statistically significant at the 5% level for technical titles and 1% level for other variables. The “natural growth” observed in untrained farmers’ human capital levels necessitates adjustment when calculating the net effect of training interventions. The post-training results demonstrate remarkable effectiveness, with over three-quarters of trained farmers utilizing WeChat groups and over three-fifths using streaming platforms for agricultural knowledge acquisition.

The human capital gap between groups widened considerably post-training. Initial differences in the four human capital variables (8.92%, 9.92%, 17.44%, and 7.40%) approximately doubled after training (15.44%, 23.01%, 43.35%, and 25.10%). The difference-in-differences analysis reveals net improvements among trained farmers: increases of 6.52% in vocational qualification certificates, 13.09% in technical titles, and 25.91% and 17.7% in WeChat and streaming platform usage respectively. T-tests confirm these net effects are significant at the 1% level.

### Difference between trained farmers and untrained farmers

The baseline human capital levels exhibit marked disparities between trained and untrained farmer groups, with trained farmers demonstrating notably higher levels. Further analysis of individual and family characteristics reveals systematic differences between the groups. Table 3 indicates that trained farmers possess significantly higher educational attainment among individual characteristics. Regarding family characteristics, trained farmers’ households show a 3 percentage point higher internet penetration rate, and their average land operation area of 76.58 mu (5.11 hectares) is approximately triple that of untrained farmers. Overall, trained farmers demonstrate superior human capital levels, better household digitalization, and larger agricultural operations. These systematic differences suggest self-selection in training participation, making direct DID analysis potentially biased. Therefore, employing PSM-DID methodology for treatment effect estimation is more appropriate to achieve unbiased analytical results.

**Table 3:** Description and difference between untrained and trained farmers (2016).

Category	Variables	Untrained group	Trained group
		Mean (SD)	Mean (SD)
Individual Characteristics	Gender (Male = 1)	0.73 (0.45)	0.76 (0.43)
	Age (Years)	45.43 (9.49)	45.42 (8.61)
	Education level (Primary school and below=1; Middle school = 2; High school = 3; Junior college = 4; Undergraduate and above=5)	2.55 (0.91)	2.71*** (0.83)
Family characteristics	Whether has internet (Yes = 1)	0.93 (0.25)	0.96* (0.19)
	Land operating area (Mu)	26.72 (40.74)	76.58*** (136.11)
Village characteristics	Distance from village to county town (Kilometers)	31.79 (19.9)	34.53 (44.53)
Provincial dummy variables	Jiangsu	0.28 (0.45)	0.29 (0.46)
	Hubei	0.32 (0.47)	0.31 (0.47)
	Heilongjiang	0.26 (0.44)	0.24 (0.43)
	Yunnan	0.15 (0.36)	0.15 (0.36)
Sample size		372	381

The asterisks on the trained group means indicate the t-statistical test significance level of the difference between the trained and untrained groups for the specific variable in 2016, ***, **, * represent 1%, 5%, 10% significance levels respectively.

### Regression results

Using PSM-DID methodology to evaluate training effects on human capital, findings demonstrate that farmer training significantly enhanced participants’ human capital (Table 4). Specifically, post-training attainment rates increased by 6.76% for national vocational qualification certificates and 13.30% for agricultural technician titles, both statistically significant at the 1% level. Regarding digital learning adoption, utilization of WeChat groups for agricultural knowledge acquisition increased by 21.03% (significant at 1%), while usage of streaming platforms like TikTok and Kuaishou rose by 11.29% (significant at 10%).

**Table 4:** Regression results from PSM-DID models.

Stage	Group	Rate of obtaining certificates or titles (%)		Rate of using network methods to learn agricultural knowledge and skills (%)	
		National vocational qualification certificate	Agricultural technician title	WeChat groups	TikTok, Kuaishou and other live streaming platforms
PSM-DID		6.76*** (2.58)	13.30*** (3.10)	21.03*** (6.48)	11.29* (6.00)
Pre-training	Untrained group C	0.31	2.1	16.86	7.97
	Trained group T	9.19	12.07	34.38	16.54
	T-C	8.88	9.97	17.53	8.57
Post-training	Untrained group C	1.95	3.5	36.51	42.35
	Trained group T	17.59	26.77	75.07	62.2
	T-C	15.64	23.27	38.56	19.85
Covariates		Yes	Yes	Yes	Yes
R-square		0.07	0.11	0.13	0.21
Sample size		1506	1506	1506	1506

Values in parentheses are robust standard errors generated by Bootstrap; ***, **, * represent significance levels of 1%, 5%, and 10% respectively.

Both descriptive and rigorous econometric analyses confirm that training programs substantially improved participants’ explicit and internalized human capital, aligning with observed training outcomes. Contemporary professional farmer training has evolved beyond singular technical instruction to encompass comprehensive knowledge, skills, and management capabilities. The curricula framework employs stratified and classified approaches, delivering specialized courses tailored to diverse agricultural sectors while incorporating essential management and professional development components. The curricula are categorized and designed according to industry type. There are specialized training programs tailored to the specific characteristics of different industries, such as crop cultivation, animal husbandry, agricultural product processing, and rural e-commerce. The curricula are also stratified according to target audience. Production- and management-oriented courses are designed for large-scale farmers and family farm owners and focus on comprehensive business management skills. Professional, skills-oriented courses target technical workers, such as farm machinery operators and plant protection specialists, and emphasize specialized skill training. Professional, service-oriented courses are designed for agricultural technology extension workers and agricultural product brokers and strengthen their service capabilities. Furthermore, the curricula are stratified by skill level. Basic training focuses on fundamental skills, intermediate training reinforces professional techniques, and advanced training emphasizes innovation and management expertise. Recent initiatives have introduced dedicated programs for new agricultural business entities and professional managers. For instance, a surveyed county’s business entity training program for macadamia nut producers integrated specialized pest control and cultivation techniques with broader competencies in brand development, e-commerce operations, and management practices, fostering comprehensive improvement in both technical expertise and managerial capabilities.

To incentivize farmers’ pursuit of comprehensive skill development and professional certification recognized by national and local authorities, thereby optimizing resource allocation and leveraging exceptional talent for rural revitalization, training programs have become increasingly integrated with certification mechanisms. For example, Shanxi Province exemplifies this integration through its “Professional Farmer Cultivation Certification Work,” which directly links training participation with skills assessment. In 2023, Shanxi Province cultivated 65,000 professional farmers, with 52,000 obtaining skill certificates, achieving an impressive 80% certification rate and substantially advancing farmers’ professional competencies. National and local policies not only promote certification pursuit but establish training participation as a prerequisite for technical title qualification. The 2022 Shijiazhuang City “Implementation Plan for Professional Title Evaluation of New Professional Farmers” mandates minimum training hours (56 for junior and 120 for intermediate titles) and offers preferential access to agricultural resources for certified farmers. Within two months of implementation, 233 farmers applied for certification, with 146 successful awards (31 junior, 115 intermediate titles). This not only fully mobilized the enthusiasm and initiative of participating farmers, but also ensured their priority rights for further participation in farmer training, helping them continuously improve their explicit human capital levels including knowledge and skills.

In addition to explicit human capital, local practices also fully demonstrate that farmer training has a promoting effect on improving farmers’ internalized human capital. Especially in recent years, with the popularization of the internet and improvements in informatization and intelligence levels, farmer training increasingly adopts online-offline integrated approaches. Internet marketing courses used in training and internet platforms such as WeChat, TikTok, and Kuaishou not only directly improve farmers’ knowledge and information reserves and expand knowledge and information source channels, but also build long-term learning and communication platforms for farmers. Compared to previously only being able to passively receive information through traditional media (television, radio, newspapers), farmers, through participating in general training and mobile application skills training, use smartphones to actively receive, select, and search for knowledge and information in more areas and content; through WeChat, TikTok, Cloud Smart Agriculture and other platforms, they visually learn agricultural production knowledge and skills, enhancing digital skills; they establish WeChat groups with instructors, classmates, and industry peers, and expanded social networks promote mutual learning, communication, and information resource sharing, thereby further improving internalized human capital, including continuous enhancement of their learning interest and learning ability.

Both descriptive statistical analysis and econometric analysis indicate that farmer training programs significantly improved participants’ human capital variable levels, achieving the original goal of farmer training policy to enhance human capital, and providing valuable human capital support for farmers to improve agricultural production efficiency and increase agricultural income.

### Balance test

To ensure result reliability, the PSM-DID implementation necessitated minimizing systematic differences in observable variables between control and treatment groups while verifying parallel trends in human capital development. This approach enables accurate measurement of training’s net impact. Balance testing results (Table 5) demonstrate successful covariate matching, eliminating systematic biases in education level, internet access, and land management area observed in Table 3. The absence of significant bias across variables strengthens the reliability of our findings.

**Table 5:** Balance test results of variables in 2016.

Variables	Untrained group C	Trained group T	T-C	t-value	P-value
Individual characteristics					
Gender (Male = 1)	0.23	0.24	0.01	0.27	0.79
Age (Years)	44.09	45.42	1.33	0.94	0.35
Education level	2.65	2.71	0.06	0.65	0.52
Household characteristics					
Internet access (Yes = 1)	0.97	0.96	−0.01	0.65	0.52
Land management area (mu)	72.47	76.58	4.11	0.15	0.88
Village characteristics					
Distance from village to county (Kilometers)	33.98	34.54	0.55	0.21	0.83
Provincial dummy variables					
Jiangsu	0.24	0.29	0.05	1.44	0.15
Hubei	0.32	0.32	0	0.04	0.97
Heilongjiang	0.29	0.24	−0.05	0.86	0.39
Yunnan	0.15	0.15	0	0.07	0.95
Sample size	312	381			

### Robustness test

We conduct DID with nearest-neighbor matching as another robustness check. The result in Table 6 is similar to the main analysis in Table 4, which demonstrates the robustness of our results.

**Table 6:** Regression results from PSM-DID models using nearest-neighbor matching.

Stage Group	Rate of obtaining certificates or titles (%)		Rate of using network methods to learn agricultural knowledge and skills (%)	
	National vocational qualification certificate	Agricultural technician title	WeChat groups	TikTok, Kuaishou and other live streaming platforms
PSM-DID	6.30*** (1.60)	12.70*** (1.96)	21.56*** (6.18)	11.37* (5.90)
Covariates	Yes	Yes	Yes	Yes
R-square	0.07	0.1	0.19	0.19
Sample size	1473	1473	1473	1473

Note: (1) Values in parentheses are robust standard errors; ***, **, * represent significance levels of 1%, 5%, and 10% respectively.

## Conclusion and policy implications

Farmer training is seen as an important way to enhance farmers’ human capital in developing countries. In recent years, China has attached great importance to farmer training work, investing more than 2 billion yuan annually to cultivate professional farmers. However, for a long time, the policy effect analysis of farmer training has been limited to agricultural production and agricultural income, while the original intention or direct purpose of the policy – improving the human capital level of participating farmers – has been largely neglected. Previous studies demonstrates that only by continuously improving farmers’ human capital levels can sustained increases in agricultural production efficiency and agricultural income be fundamentally achieved. This study uses nationally representative survey data and employs the PSM-DID research method to examine the impact of farmer training on participants’ human capital. Empirical analysis finds that farmers who participated in training showed significant improvements in explicit human capital levels, with the rate of obtaining national vocational qualification certificates increasing by 6.76% and the proportion obtaining agricultural technician titles increasing by 13.30%. Meanwhile, internalized human capital levels also improved, with the rates of using online means to learn agricultural knowledge and skills significantly increasing – learning through WeChat groups increased by 21.03%, and learning through live streaming platforms like TikTok and Kuaishou increased by 11.29%. These findings indicate that farmer training effectively fulfills its fundamental policy objective by enhancing both dimensions of human capital, suggesting that investments in agricultural education yield direct returns in farmer capability development.

The policy implications of this study are twofold: First, greater emphasis should be placed on farmers’ human capital levels, particularly for professional farmers who represent the advanced force of China’s agricultural productivity yet still lag behind their counterparts in developed countries. Only by accelerating improvements in China’s professional farmers’ human capital can we fundamentally enhance national agricultural competitiveness. Second, farmer training policies should prioritize human capital enhancement as both means and goal, moving beyond purely utilitarian approaches to focus on sustainable capability building that drives long-term productivity gains. Special attention should be given to developing farmers’ internalized human capital, particularly online learning abilities, as these implicit capabilities become increasingly crucial in an era of rapid agricultural technology iteration and digital integration. For instance, we could establish a blended “online + offline” personalized learning system that designs differentiated digital learning paths for farmers of various ages and educational backgrounds. A three-tiered learning network of “experts-farmers-peers” can be built using WeChat groups to establish a regular Q&A mechanism that encourages farmers to proactively ask questions and share experiences. Short video platforms, such as Douyin, can be used to create contextualized teaching materials that deliver precise knowledge according to the agricultural season. These materials can dynamically adjust content recommendations based on farmers’ learning feedback. This will form a personalized learning ecosystem and enhance farmers’ independent learning abilities in the digital age.

This study has several limitations. The sample is limited to four provinces, and the generalizability of the results needs further verification. The long-term sustainability of the training effect is not be assessed. Future research could extend the study period to obtain a more comprehensive evaluation of training effectiveness.
